# Omnidirectional Sensor Design for Distributed Laser Measurement Systems

**DOI:** 10.3390/s24030961

**Published:** 2024-02-01

**Authors:** Fei Liu, Qing Liu, Yaohui Zhi, Ting Shang

**Affiliations:** 1 School of Materials Science and Engineering, Xi’an University of Science and Technology, Xi’an 710054, China; liufei@stu.xust.edu.cn; 2 School of Automation and Information Engineering, Xi’an University of Technology, Xi’an 710048, China; st107@xaut.edu.cn; 3 School of Mechanical and Precision Instrument Engineering, Xi’an University of Technology, Xi’an 710048, China; 2210320117@stu.xaut.edu.cn

**Keywords:** distributed laser measurement system, omnidirectional sensor, signal detection, precision measurement

## Abstract

Distributed laser measurement systems, widely used in high-end equipment such as airplanes, ships, and other manufacturing fields, face challenges in large spatial measurements due to laser plane obstructions and weak intersections. This paper introduces a novel omnidirectional sensor with enhanced adaptability to complex environments and improved measurement accuracy. Initially, an integrated omnidirectional measurement model is established, followed by the analysis of the optical path of the front-end detector, and the design of a signal-conditioning circuit for the photoelectric conversion of the front-end laser signal, Subsequently, a circuit testing platform is established to validate the detection functionality, and the corresponding results indicate that the symmetry of the output waveform is under 10 ns, the response time is under 100 ns, and the maximum detection distance is 22 m. Further, experimental results demonstrate the superiority of omnidirectional sensors over planar ones in complex environments, successfully receiving 360° laser signals. The positional accuracy of the common point to be measured on the top of the omnidirectional sensor is confirmed to exceed 0.05 mm, and the accuracy of the angle of attitude exceeds 0.04°. Using the laser tracker, the measurement accuracy of the system is verified to be better than 0.3 mm. When rotating in the horizontal and pitch directions, the measurement accuracy is better than 0.35 mm and 0.47 mm, respectively, fulfilling the sub-millimeter precision requirement and expanding the application scope of distributed laser measurement systems.

## 1. Introduction

A distributed laser measurement system, based on the principle of forward spatial angle intersection, is essential in precision manufacturing sectors like aerospace and large-scale shipbuilding [1]. The system is known for millimeter and sub-millimeter levels of high-precision measurement capabilities [2], integrating multiple devices for target measurement and localization. It stands out for its high measurement accuracy, strong interactivity, wide measurement range, and strong field practicability [3]. Within this system, the photoelectric sensor plays a crucial role, directly influencing the measurement range and accuracy [4]. These sensors not only receive optical signals, but also adapt to various measurement environments, performing functions like information conversion, extraction, and calculation. However, spatial obstructions in complex environments limit the field-of-view angle of the existing planar-type sensors, necessitating full-view angle reception for navigation [5]. Therefore, developing omnidirectional sensors for distributed laser measurement systems is vital to address these challenges and enhance the system’s overall effectiveness.

Photoelectric sensors can be categorized into three types based on the measurement context: planar photoelectric sensors, full-range photoelectric sensors, and combined sensors [6,7,8]. Planar-type photoelectric sensors are noted for their high measurement accuracy and simple mechanical structure, giving them the lowest design difficulty and the widest range of applications [9]. Notable implementations include Nikon’s iGPS system in Japan [10] and Tianjin University’s wMPS system [11]. However, due to their structural limitations, planar photoelectric sensors typically have a measurement angle of less than 120°, restricting the system’s measurement range. To address this, Nikon developed a new full-range photoelectric sensor that uses multiple rectangular silicon photocells arranged in a certain pattern. These sensors create a light-sensitive surface resembling a cylindrical surface, but still lag behind planar photoelectric sensors in measurement accuracy. To solve the limitation of both planar and full-range sensors, particularly in measuring the surface coordinates of objects, companies like Leica and Nikon have developed combined sensors [12]. In China, institutions such as Tianjin University and Xi’an Jiaotong University have carried out theoretical research on these combined sensors [13,14]. For instance, Jianwei Wu et al. from Xi’an Jiaotong University have designed array-type and combined-type photoelectric sensors. However, the overall measurement accuracy of these designs still needs improvement.

This paper addresses the challenge where front-end sensors in existing distributed laser measurement systems struggle to simultaneously meet the dual demands of a wide reception range and high measurement accuracy, especially when laser signals are obstructed in a complex measurement environment. To tackle this issue, this paper focuses on designing an omnidirectional sensor by establishing an integrated measurement model for the sensor, conducting optical circuit analysis, and designing a circuit for photoelectric conversion signal conditioning. Finally, an experimental platform is set up to validate the omnidirectional sensor’s signal reception range and measurement accuracy.

## 2. Principle of Omnidirectional Sensor Measurement

**Figure 1:**
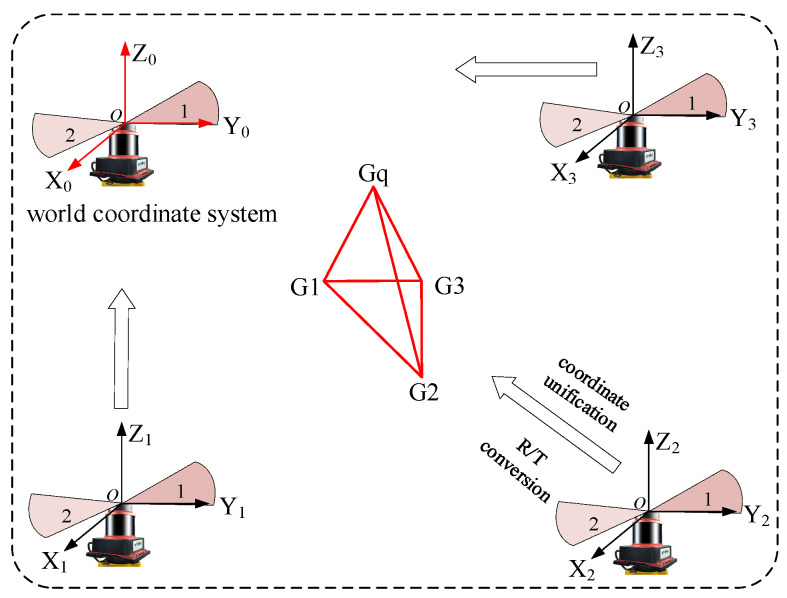
Omnidirectional sensor measurement model.

In this work, the design of a 16-plane omnidirectional sensor with symmetric top and bottom structures, tailored for the distributed laser measurement system, is presented. This design is influenced by challenges such as field occlusion and the limitation of the sensor’s receiving field of view. In practical application, an omnidirectional spatial measurement method can be established by processing the measurement information of a minimum of three photodetection nodes, along with structural distance parameter data. As illustrated in Figure 1, the design utilizes a partial octahedral model to establish an omnidirectional measurement model within a distributed laser measurement system.

In this design, *G*_1_, *G*_2_, etc., are the individual detection points on the sensor and *G_q_* is the common point to be measured, which can be obtained based on the structural parameter *D_sx_* of the omnidirectional sensor and the principle of spatial laser rendezvous.

(1) $$\left\{\begin{array}{c} L_{1}=A_{K1s}X_{s}+B_{K1s}Y_{s}+C_{K1s}Z_{s}=0\\ L_{2}=A_{K2s}X_{s}+B_{K2s}Y_{s}+C_{K2s}Z_{s}=0\\ {\left\|G_{s}-G_{x}\right\|}_{2}=D_{sx} \end{array}\right.$$

Additionally, $\left(A_{K1s},B_{K1s},C_{K1s}\right)$ and $\left(A_{K2s},B_{K2s},C_{K2s}\right)$ denote the parameters of the laser plane; $\left(X_{s},Y_{s},Z_{s}\right)$ denotes the spatial coordinate value of each detection point; s,x are the number of the receiving nodes; $D_{sx}$ is the distance parameter between the detection points s,x; $G_{s},G_{x}$ signifies the distribution of detection points on the omnidirectional sensor; and $L_{1},L_{2}$ represent the two sector equations for the transmitting base station. As the measurement information of at least three detection nodes is required, a nonlinear optimization algorithm is employed to solve it. Given the objective optimization function $F_{\min}$, the optimal solution is attainable when $F_{\min}$ tends to infinity.

(2) $$F_{\min}=\sum_{s=1}^{n}\left(L_{1}^{2}+L_{2}^{2}\right)+\sum_{s=1}^{n}\sum_{x=1}^{n}{\left\|G_{s}-G_{x}\right\|}_{2}^{2}$$

To determine the spatial coordinates of the target to be measured, it is essential to solve the common point to be measured, $G_{q}$, on the omnidirectional sensor, and the spatial coordinates of the detection nodes can be obtained through Equation (2); subsequently, the spatial coordinates of the common point to be measured can be determined using the distance parameter between $G_{q}$ and the detection node $G_{sx}$.

(3) $${\left\|G_{sx}-G_{q}\right\|}_{2}=D_{sxq}$$

where $D_{sxq}$ is the distance parameter between $G_{q}$ and the probe node $G_{sx}$.

Attitude measurement is achieved through the transformation of the coordinate system in response to variations in the omnidirectional sensor’s attitude. This involves selecting three of the detection nodes to establish the omnidirectional coordinate system and establishing the global coordinate system for the distributed laser measurement system. The alignment of these two coordinate systems is facilitated by employing rotational translation relationships, resulting in their unification under a common coordinate system.

(4) $$\left\{\begin{array}{c} {\left[x_{si},y_{si},z_{si}\right]}^{T}=R_{W}{\left[x_{gi},y_{gi},z_{gi}\right]}^{T}+T_{W}\\ E_{\min}={\sum_{i=1}^{s}\left(R_{W}{\left[x_{gi},y_{gi},z_{gi}\right]}^{T}+T_{W}-{\left[x_{si},y_{si},z_{si}\right]}^{T}\right)}^{2} \end{array}\right.$$

Here, $\left(x_{si},y_{si},z_{si}\right)$ and $\left(x_{gi},y_{gi},z_{gi}\right)$ represent the coordinates in the sensor coordinate system and the world coordinate system, respectively. Additionally, $R_{W}$ and $T_{W}$ denote the rotation and translation matrices that are unified for both coordinate systems.

In Equation (4), there exist two matrices with six unknowns, necessitating the knowledge of at least three or more coordinates of the probe nodes for its resolution. Consequently, the solution process can be reformulated as a nonlinear optimization problem.

When the omnidirectional sensor undergoes random attitude changes in space, the coordinates are recorded at the initial attitude position. Utilizing the rotational translation relationship derived from Equation (4), the randomly altered attitudes can be standardized to the initial moment. Subsequently, the spatial coordinates of the omnidirectional sensor after the attitude change can be determined.

## 3. Design of Omnidirectional Sensor

### 3.1. Optical Path Analysis

The distributed laser measurement system needs to convert all of the photoelectric information received by the photoelectric sensor into time information [15], and then convert the time information into angle information. This process is called *T*–*A* conversion, where ω denotes the rotational speed of the transmitting base station and *n* denotes the laser sector:

(5) $$\theta_{n}=\omega \cdot (t_{n}-t_{0})$$

During the conversion process, it is essential to maintain the constant position of the extracted feature points and achieve high time extraction accuracy [16]. This necessitates the convergence of the received fan-shaped laser to converge to the center of the silicon photocell. Therefore, the optical path of the omnidirectional sensor is designed to realize the omnidirectional reception of spatial laser signals using a special reflecting prism combined with a photoelectric conversion circuit. 

**Figure 2:**
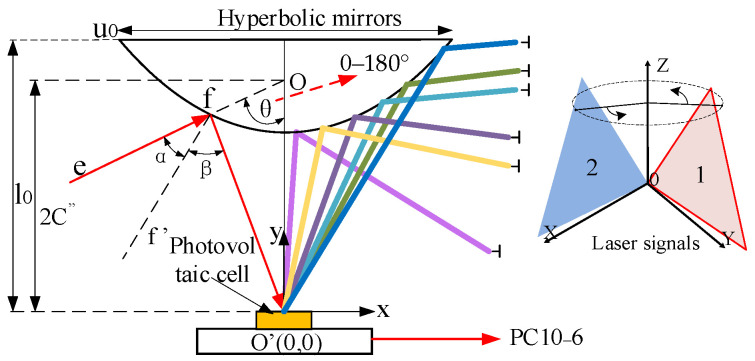
Hyperbolic light path reflection model.

When the scanning laser signals emitted by the transmitting base station intersect with the hyperbolic reflecting prism, the incident light from each direction converges at the center of the silicon photocell at *O’* after reflection. This process outputs an electric pulse signal through the photoelectric converter circuit. Simultaneously, the extension lines of the incident light converge at the common point *O*. The hyperbolic light path reflection model is illustrated in Figure 2.

The focal point *O’* of the hyperbolic mirror is set at the position of the center of the silicon photocell circle *O’*, and the incident line *ef* intersects the other focal point *O* of the hyperbolic lens. The coordinates of the point of incidence of the light are $f=(\frac{u_{0}}{2},l_{0})$, the diameter of the hyperbolic mirror is *u_0_*, and *l*_0_ denotes the distance from the center of the silicon photocell circle to the center of the top of the hyperbolic mirror, and the simplified mathematical model of the hyperbolic reflector is:

(6) $$\frac{{\left(y-C^{\prime \prime }\right)}^{2}}{A^{\prime \prime 2}}-\frac{x^{2}}{B^{\prime \prime 2}}=1$$

where $A^{\prime \prime },B^{\prime \prime }$ represent the parameters of the equation of Equation (6); $C^{\prime \prime }$ represents the focal length; $C^{\prime \prime }=\sqrt{A^{\prime \prime 2}+B^{\prime \prime 2}}$; and the focal length $OO^{\prime }=2C^{\prime \prime }$. The above parameter values are substituted into Equation (1) to further obtain the system of equations.

(7) $$\left\{\begin{array}{c} \frac{l_{0}-\sqrt{A^{\prime \prime 2}+B^{\prime \prime 2}}}{A^{\prime \prime 2}}-\frac{{u_{0}}^{2}}{4B^{\prime \prime 2}}=1\\ \frac{l_{0}-2C^{\prime \prime }}{\frac{u_{0}}{2}}=\tan\left(\frac{\pi }{2}-\theta \right) \end{array}\right.$$

This paper, synthesizing theoretical analysis with practical tests, selects First Sensor’s PC10-6 model. The theoretical model for the optical path reflective sensor is designed to ensure that the incident light signal is reflected to the center of the circular area of the silicon photocell, maintaining the integrity of the signal output waveform. This design facilitates the omnidirectional 360° refraction of the signal to the detector when the receiving angle is horizontally aligned with the light source. However, challenges emerge when the incident angle of the light source is in the longitudinal direction and is less than 90°, leading to excessive angle deviation that hinders the laser beam from being refracted to the central position of the detector. To address this, the assembly process requires precise and complex tooling to ensure the accurate targeting of the refracted focus of the optical path. 

### 3.2. Photoelectric Conversion Circuit Design

The omnidirectional sensor plays a critical role as a primary acquisition component for optical signals in the distributed laser measurement system. Its primary function is to convert the analog laser signal within the measurement field into a pulsed digital signal. This digital signal is required to maintain a high signal-to-noise ratio and preserve excellent waveform quality. Achieving these objectives necessitates the design of a signal conditioning circuit responsible for extracting the light source signal from the base station and processing it through multiple stages, including amplification, analog-to-digital conversion, and noise filtering. These stages are essential to ensure the measurement accuracy of the system. The comprehensive design scheme for this circuit is presented as follows.

The preamplifier circuit represents the first stage amplifier section. It is instrumental in amplifying, filtering, and processing the signal for subsequent stages. Its performance significantly impacts factors such as the signal-to-noise ratio and sensitivity. To minimize the high-speed pulse signal distortion in the design, the preamplifier must have enough signal bandwidth *f*; this is achieved by integrating a small capacitor *C_f_* in parallel on both sides of the feedback resistor *R_f_* to form a trans-impedance amplifier, providing the circuit with a negative feedback function. The output voltage of the trans-impedance amplifier can be calculated using the following formula:

(8) $$U_{o}=-R_{f}I$$

To prevent excessively large values due to the feedback resistor, its resistance value can be determined based on the output voltage amplitude. The feedback resistor and feedback capacitance work in tandem to limit the signal bandwidth. The capacitance of the feedback capacitor is determined by the amplifier bandwidth and feedback resistor, as expressed by the following formula:

(9) $$1/(2\pi R_{f}C_{f})=\sqrt{GBP/(4\pi R_{f}C_{D})}$$

where *GBP* denotes the operational amplifier bandwidth and *C_D_* denotes the silicon photocell junction capacitance.

After the (*I*/*V*) conversion of the preamplifier circuit, the signal strength is still weak, so it is necessary to add an inverse-proportional amplifier circuit for the secondary amplification, in which the inverse-proportional amplified output signal can be calculated.

(10) $$V_{OUT}=-\frac{R_{f}}{R_{s}}V_{IN}$$

The silicon photocell, task with receiving laser signals, is significantly influenced by environmental factors such as fluctuating light sources, laser diffuse reflection, stray light incidence, temperature and humidity variations, and electromagnetic interference.

In indoor measurements, the majority of ambient light can be effectively filtered out using an 850 nm bandpass filter. However, periodic noise interference persists in the signal, and this interference is subsequently eliminated through the application of a voltage reference chip. This process facilitates the modulation of the front-end laser signal. The pre-stage analog signal, after undergoing multi-stage amplification and conditioning, requires conversion to a pulse signal for collection. The design incorporates a hysteresis comparator circuit, wherein the hysteresis voltage parameters are carefully chosen to ensure stable output even in the presence of spurious signal interference.

**Figure 3:**
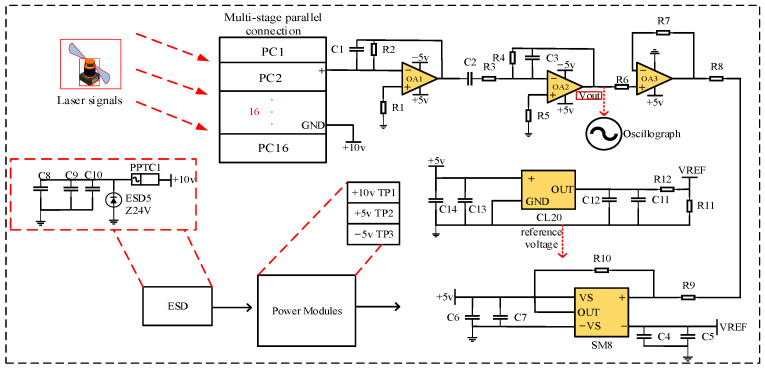
Signal conditioning circuit test system.

Upon finalizing the overall circuit schematic design, the circuit test is established, as shown in Figure 3. 

**Figure 4:**
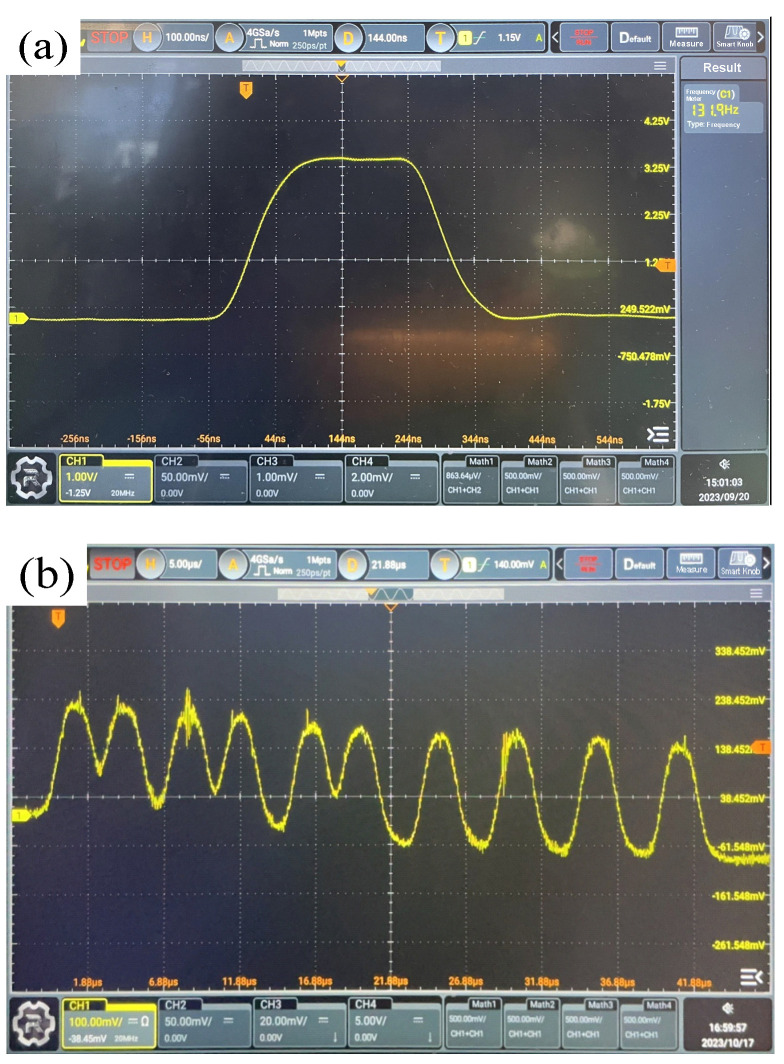
(**a**) Single-board photoelectric signal output waveform. (**b**) Photoelectric signal output waveform after multi-stage parallel connection.

This system integrates all of the components listed in Table 1, enabling the functional evaluation of the sensor circuits. The signal waveforms of the circuit at the secondary output are scrutinized using an oscilloscope for both a single silicon photocell and 16 silicon photocells in cascaded configuration, as depicted in Figure 4a,b.

The findings reveal that the response time of the former is below 100 ns, with waveform symmetry within 10 ns. The signal-to-noise ratio, measured at approximately 60 dB, surpasses the stipulated design requirement of over 20 dB. Notably, the comprehensive waveform’s response speed remains uncompromised even after cascading. The voltage amplitude is intricately linked to both the light-receiving area of the detection node and the distance between the sensor and the base station. Throughout comprehensive testing, the sensor-to-base station distance is notably extensive, leading to a substantial reduction in amplitude. The test outcomes confirm that the design adheres meticulously to the measurement principles of the distributed laser measurement system. Specifically, it involves the extraction of midpoint positions from the rising and falling edges of the output pulse signal for subsequent calculation.

**Figure 5:**
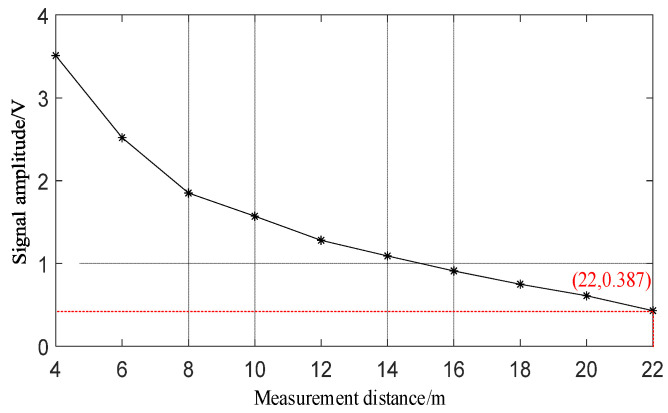
Signal measurement distance results.

The laser signal’s measurement distance is systematically evaluated over a range from 4 m to 22 m from the illumination source. During the experimental stage, the separation between the silicon photocell and the light source is meticulously adjusted. The resulting correlation between the amplitude of the measurement signal and the distance is illustrated in Figure 5. Notably, with increasing distance, there is a rapid decline in signal amplitude. Additionally, considering the threshold level of the high-speed comparison circuit at approximately 300 mV, it is determined that pulse signals could be reliably output within a range of 22 m. Consequently, the circuit has been meticulously designed to accommodate and optimize practical usage within this operational range of 22 m.

**Figure 6:**
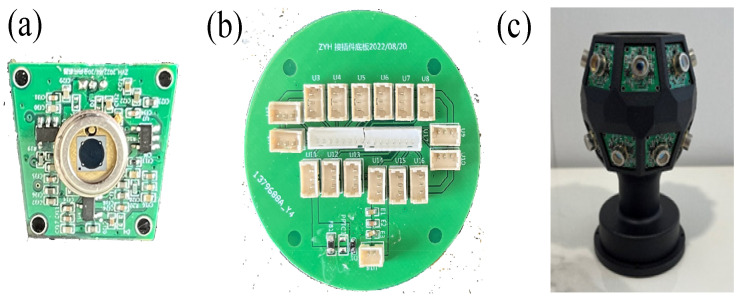
(**a**) Photoelectric sensor. (**b**) Adapter plate. (**c**) Omnidirectional sensor.

Upon the successful completion of circuit function verification, subsequent tasks encompassed layout and wiring, electrical rule checking, and additional procedures to finalize the PCB for both the omnidirectional sensor and adapter board, as depicted in Figure 6a,b. Integrating insights from the omnidirectional measurement model, optical path analysis, and the circuit design, the top and bottom symmetry of a 16-sided structure is employed. Each of the 16 faces represents an individual receiver node. The design incorporates a multi-stage parallel connection involving 16 receiver nodes through the adapter board, detailing the specific structure in Figure 6c. As delineated by the optical path analysis in Section 3.1, the surface encapsulation of the silicon photocell for each detection node will feature a specialized reflective prism. This prism is adept at expanding the laser signal reception range of the entire sensor.

**Figure 7:**
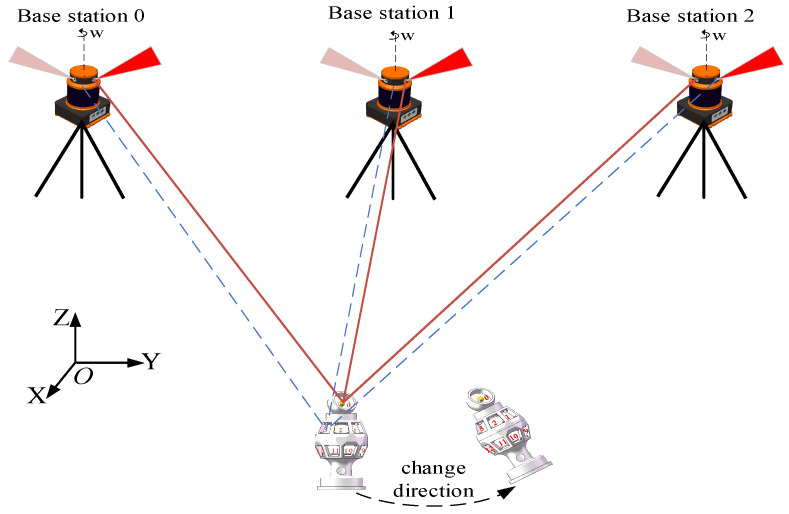
Rapid calibration model.

The calibration model is illustrated in Figure 7. Following the physical fabrication, it is necessary to calibrate the distances between adjacent sensing nodes on the sensor and between the nodes and their top measurement head (common test point). In the structural design, a spherical socket is reserved at the top to accommodate a spherical sensor as the measurement head. Subsequently, with the assistance of a distributed laser measurement system, the internal parameters are calibrated by rotating the spherical sensor during each measurement, orienting it towards the transmitting base station. By measuring the spatial coordinates in the world coordinate system between each pair of 16 detection nodes and between each node and the measurement head, the required distance values can be calculated. Distances for non-adjacent nodes are determined through spatial vector relationships.

## 4. Experiment and Discussion

To validate the practicability of the omnidirectional sensor proposed in this study within a distributed laser measurement system, experiments were conducted using four transmitting base stations. These stations operated at rotational speeds of 2750 r/min, 2800 r/min, 2975 r/min, and 3000 r/min, respectively. A single base station achieved measurements up to 30 m. The setup included two near-infrared lasers with a wavelength of 850 nm, a pupil power of 30 mW, and a sector angle of 120°, mounted on a rotating head. Additionally, a planar receiver, a high-speed processor, and an upper computer were employed to construct an experimental verification platform for distributed laser measurement systems, assessing the signal reception range and measurement accuracy.

### 4.1. Signal Reception Range Experiment

**Figure 8:**
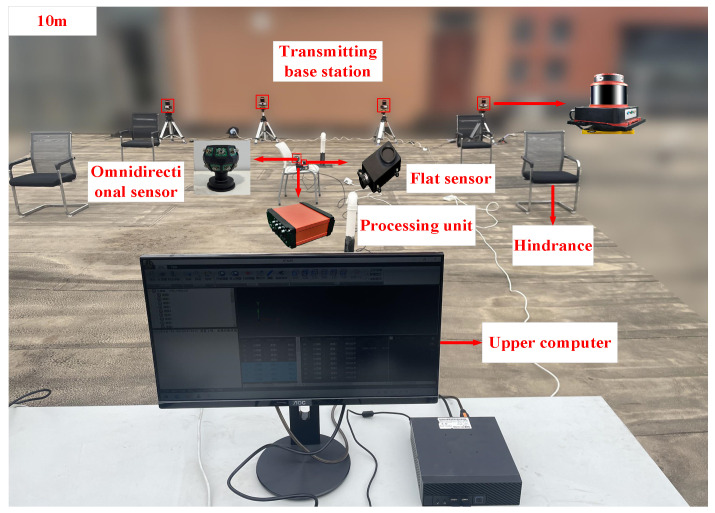
Signal reception range test experiment.

**Figure 9:**
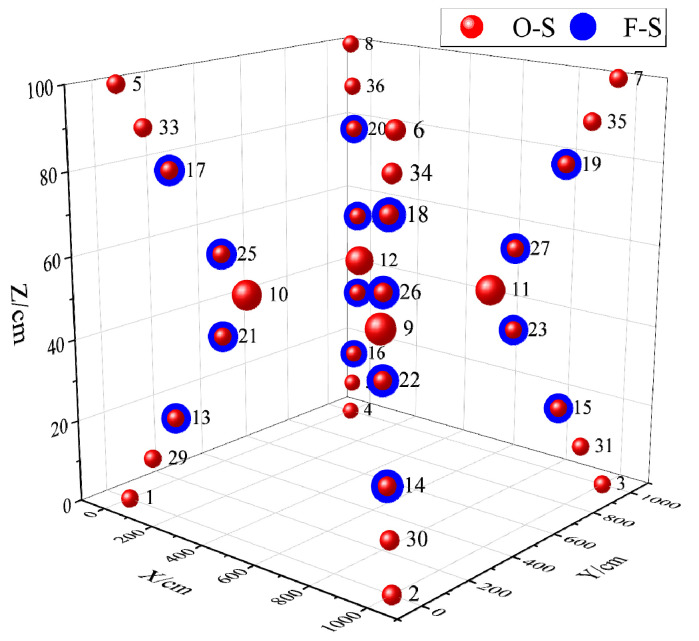
Experimental results of signal reception range of two sensors.

As illustrated in Figure 8, a spatial area with dimensions of 10 m × 10 m × 2 m was established within the measurement site. This area included the placement of four obstacles to facilitate the selection of suitable measurement points. Subsequently, both the omnidirectional and planar sensors were individually relocated to the chosen measurement points. The data output was then monitored using the upper computer software, and the resulting measurement outcomes are presented in Figure 9. To validate the experimental precision, six representative points with no output were specifically chosen. The output voltages were measured using a voltmeter, with detailed results provided in Table 2.

The experimental results highlighted a significant difference in performance between the planar and omnidirectional sensors. Specifically, the planar sensor fails to output data when the target points are located at 1–8, 9–12, and 29–36. This lack of output is attributed to the limited laser signal reception range of the planar sensor, especially at peripheral points and obstructions at the middle points. Conversely, the omnidirectional sensor consistently produced data output across all tested points. To further assess the accuracy, the output voltages of six of the no-signal points were tested using a voltmeter. Corresponding findings revealed that at four points where the planar sensor was located, the output voltage was zero. At the remaining two points, there was no data output due to the output voltages being significantly below the 300 mv threshold level. These results validate the design and functionality of the omnidirectional sensor developed in this paper. It demonstrates a marked improvement in the reception range of the laser signal and shows adaptability in complex measurement environments.

### 4.2. Measurement Accuracy Experiment

**Figure 10:**
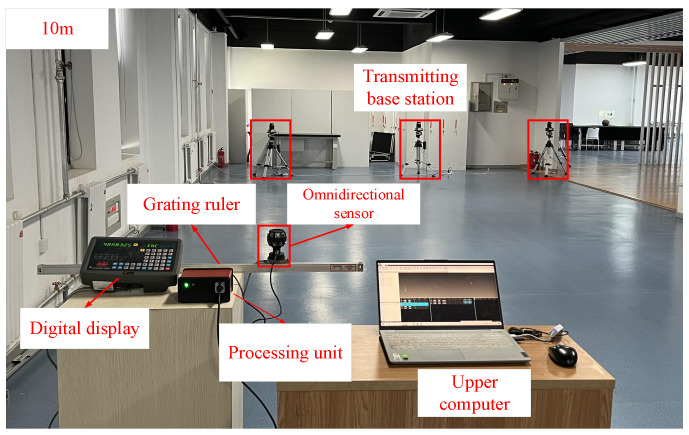
System accuracy test experiment.

This section focuses on verifying the measurement accuracy and stability of the omnidirectional sensor in a distributed laser measurement system. This is achieved by setting up a specific measurement field, as shown in Figure 10. The measurement range is 10 m × 7 m × 2 m. To ensure accurate calibration and validation, a calibration bar traverses this measurement field multiple times. This process is essential for calibrating the outer parameters of the transmitting base station and for establishing a global coordinate system.

**Figure 11:**
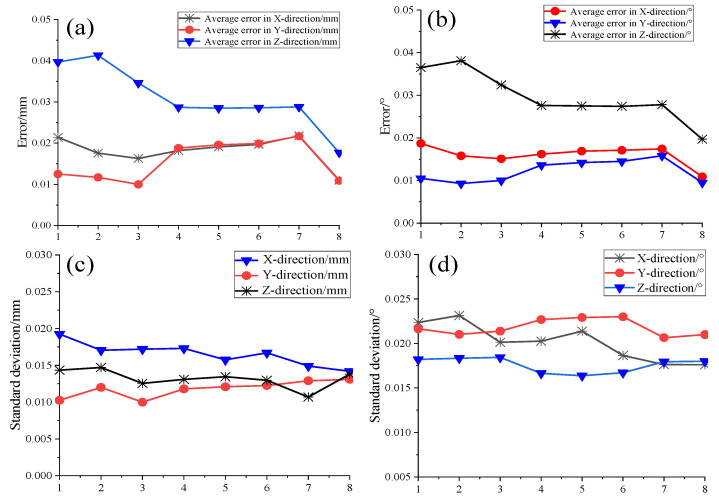
(**a**) Omnidirectional sensor three-axis position measurement result. (**b**) Three-axis attitude angle measurement result. (**c**) Position measurement standard deviation. (**d**) Attitude angle measurement standard deviation.

To validate the measurement accuracy of the targets, the initial step involves verifying the positional accuracy of the virtual common point positioned atop the omnidirectional sensor. The spatial coordinates of this point were initially determined using a distributed laser measurement system. Subsequently, these coordinates were then processed using an omnidirectional position-solving algorithm, which incorporates angular information data. To assess the experiment’s accuracy, the sensor was rotated every 45° along the 360° circle direction for a total of eight rotations, with each position undergoing 200 repetitions to calculate the average measurement error. The accuracy of the attitude angle was examined by altering the sensor’s orientation and repeating the aforementioned operation. The results are presented in Figure 11a,b, while the standard deviation of the two sets of test data was computed, and the outcomes are illustrated in Figure 11c,d.

The outcomes indicate that, by altering the position and attitude of the omnidirectional sensor and conducting 200 repetitions of measurements on its virtual common point, the position repeatability error is maintained at less than 0.05 mm. Additionally, the three-axis attitude angle repeatability error is kept under 0.04°, and the measured data exhibit minimal dispersion, demonstrating excellent stability.

**Figure 12:**
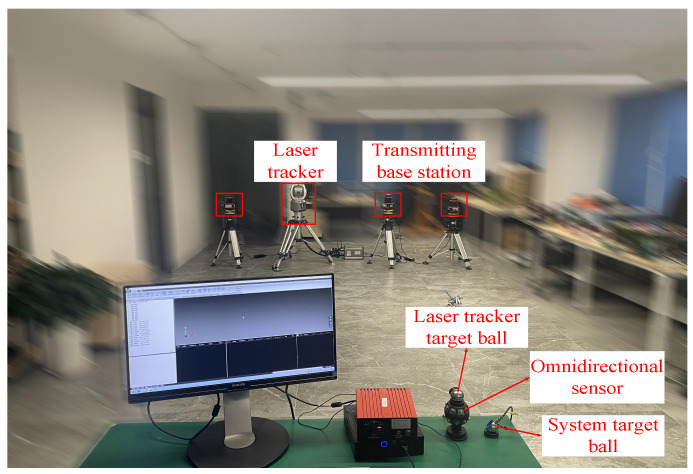
Absolute accuracy verification of the omnidirectional sensor.

Subsequently, the Faro laser tracking system was employed to perform absolute accuracy assessments on the omnidirectional sensor. The measurement field, as depicted in Figure 12, was established with dimensions of 8 m × 5 m × 2 m.

**Figure 13:**
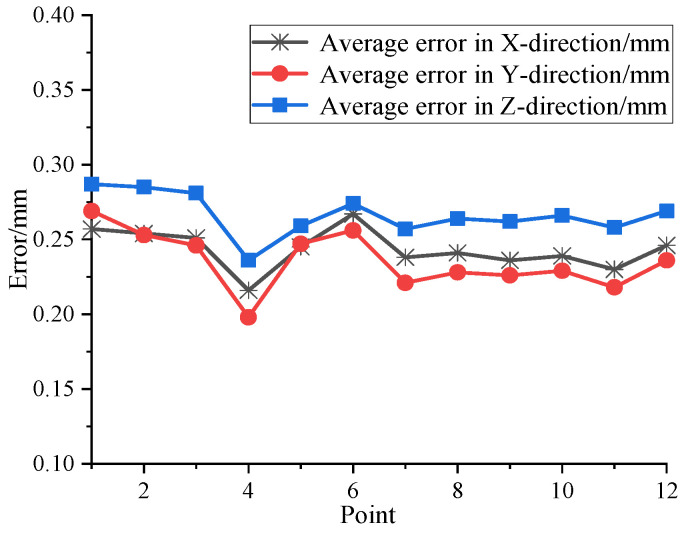
Omnidirectional sensor accuracy measurement results.

The centers of the spherical sensors in both systems are aligned. Through the measurement of common points and the application of rotation and translation relationships, the two systems could be standardized to the same coordinate system. Following coordinate standardization, 12 random positions were designated in the measurement field. The laser tracker target ball was inserted into the socket of the omnidirectional sensor and repositioned to the specified locations. The spatial coordinates of each point were individually measured by the two systems and then standardized to the laser tracker system. This entire process was replicated 200 times at each location. The comparison of the measurement results from the two sensors is depicted in Figure 13.

The results indicate that the maximum measurement error of the omnidirectional sensor’s common test point is within 0.3 mm. This level of precision meets the sub-millimeter precision requirements for distributed laser measurement systems in multi-station convergence measurement scenarios.

**Figure 14:**
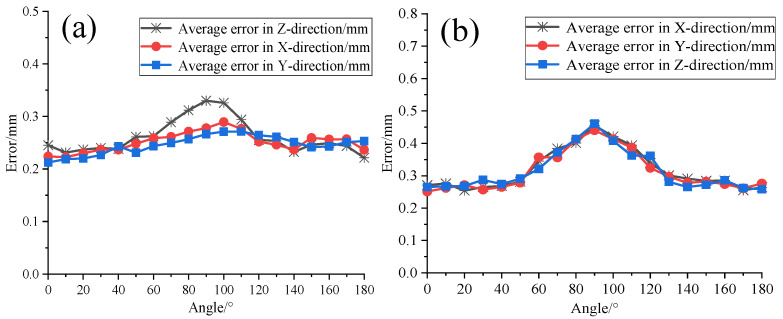
(**a**) Horizontal three-axis measurement error. (**b**) Pitch three-axis measurement error.

Based on the above accuracy verification, further validation of the measurement errors of the system was conducted when the sensor rotated in the horizontal and pitch directions. The laser tracker target ball was fixed in its socket, and rotation measurements were performed at 10° intervals in each direction. During each measurement, the target ball was rotated to face the transmitting base station. Measurements were conducted 200 times at each position. A comparison of the measurement results for the two sensors is shown in Figure 14.

The results indicate that when the omnidirectional sensor rotates in the horizontal direction, the maximum error is within 0.35 mm. In the pitch direction, specifically from 60° to 120°, there is a slight decrease in accuracy, with the maximum error still within 0.47 mm, still meeting the sub-millimeter measurement requirements of the system. This further validates that the omnidirectional sensor designed in this study exhibits excellent measurement accuracy and stability.

### 4.3. Experimental Discussion

The experimental investigation focused on the signal-receiving range and measurement accuracy of the omnidirectional sensor. Within the measurement field, appropriate measurement points were established, and both the planar sensor and the omnidirectional sensor were relocated to these points for testing. As the operational range of the two types of sensors expanded and laser signal obstructions were introduced, the omnidirectional sensor consistently received signals and provided output, whereas the planar sensor exhibited limited output within a specific range. Subsequently, six representative points with no output were examined using a voltmeter. Among these, four points associated with the planar sensor displayed an output voltage amplitude of 0, and the output voltage of the two obstacle points was significantly below the 300 mV threshold level of the high-speed comparison circuit. These findings further substantiate the precision of the omnidirectional 360° laser signal reception.

Before the practical test, the position and attitude accuracy of its top virtual common point was verified by repeating the test 200 times. The position accuracy of the common point was within 0.05 mm, and the accuracy of the three-axis attitude angle was within 0.04°, demonstrating good stability. The absolute accuracy of the omnidirectional sensor was verified using a laser tracking system in conjunction with a distributed laser measurement system and an omnidirectional position-solving algorithm. Measurements were conducted at 12 different positions, each repeated 200 times, and the comparison revealed a maximum error of less than 0.3 mm. Subsequently, the system’s measurement accuracy was further validated during horizontal and pitch rotations, with maximum errors less than 0.35 mm and 0.47 mm, respectively, meeting the sub-millimeter requirements of distributed laser measurement system indicators.

## 5. Conclusions

This paper presents a comprehensive design of an omnidirectional sensor within a distributed laser measurement system. It introduces an omnidirectional measurement method based on the intersection of front-end laser scanning, addressing key aspects such as the design of the optical path of the omnidirectional sensor and the development of a photoelectric conversion circuit for front-end laser signal conditioning. The practicality of this omnidirectional sensor is validated through the construction of the experimental platform of the distributed laser measurement system, confirming its capability to accurately receive 360° laser signals in complex environments. This advancement is particularly significant for addressing the issue of weak intersections caused by laser plane obstruction in large space measurements. The successful implementation of the proposed omnidirectional sensor marks a notable improvement in distributed laser measurement systems, enhancing their accuracy and reliability in diverse and challenging measurement scenarios.

## Figures and Tables

**Table 1:** Component model/value.

Component	Model/Value
PC	PC10-6
OA	OP065, OP084, OP083
TP	TPS4701, TPS3301
SM8	SGM874
CL20	CLREF2018
R1, R2, R3, R4, R5, R6, R7, R8, R9, R10, R11	24 kΩ, 1.5 kΩ, 33 kΩ, 1.7 kΩ, 100Ω, 75 kΩ, 60 Ω, 10 kΩ, 75 Ω, 75 10 ppm, 75 10 ppm
C1, C2, C3, C4, C5, C6, C7, C8, C9, C10, C11, C12, C13, C14	1.5 pF, 470 nF, 1 pF, 10 uF/5 V, 0.1 uF/50 V, 10 uF/10 V, 0.1 uF/50 V, 10 uF/16 V, 10 uF/50 V, 0.1 uF/50 V, 22 uF/16 V, 0.1 uF/50 V

**Table 2:** Comparison of output amplitude of two sensors.

Point	O-S/mv	F-S/mv
4	2036.46	0
5	2476.41	16.65
10	3983.24	0
11	3214.93	0
34	2827.80	32.52
35	3106.00	0

## Data Availability

Data are contained within the article.
